# Altered regional gray matter volume in Chinese female patients with bulimia nervosa

**DOI:** 10.1186/s12888-020-02493-4

**Published:** 2020-03-02

**Authors:** Xiao Li, Xiaowei Liu, Yu Wang, Lingfei Li, Linli Zheng, Yaya Liu, Jing Ma, Lan Zhang

**Affiliations:** 1 Mental Health Center, West China Hospital, Sichuan University, No. 28 Dian Xin Nan Road, Chengdu, 610041 Sichuan China; 2 Zun Yi Psychiatric Hospital, Zunyi, 563000 Guizhou China

**Keywords:** Bulimia nervosa, MRI, Grey matter volume, Voxel-based morphometry

## Abstract

**Background:**

Bulimia nervosa (BN) is a psychiatric disorder with unclear pathophysiology. Several studies have associated BN with structural and functional changes in the brain, but findings have been inconsistent. Here we explored this potential association in a small group of Chinese women with BN.

**Methods:**

This retrospective study examined 34 women with BN and 34 age-matched healthy controls, all of whom underwent T1-weighted magnetic resonance imaging (MRI). Voxel-based morphometry was carried out to explore alterations in regional grey matter volume (GMV) that may be associated with BN.

**Results:**

The BN group showed smaller GMV in the left medial superior frontal gyrus (SFGmed.L), right superior temporal gyrus (STG.R), right median cingulate and paracingulate gyri (DCG.R), left median cingulate and paracingulate gyri (DCG.L) and left dorsolateral superior frontal gyrus (SFGdor.L). No regions showing GMV increases in BN were identified. The GMV reduction did not correlate with body mass index, duration of illness, or patients’ self-esteem or overall self-evaluation. GMV reduction correlated negatively with age in the SFGmed. L (r = − 0.516, *P* < 0.005), DCG. R (r = − 0.556, P < 0.005), DCG. L (r = − 0.576, *P* < 0.05) and SFGdor. L (r = − 0.576, P < 0.005).

**Conclusions:**

Women with BN show reduced GMV in several brain regions, but it is difficult to know whether these changes are the result of BN pathology or of binge-eating and compensatory behavior. These changes may be associated with impaired inhibitory control, body dissatisfaction and emotion dysregulation.

## Background

Bulimia nervosa (BN) is an eating disorder characterized by recurrent episodes of binge eating and inappropriate compensatory behaviors to prevent weight gain [1]. It usually occurs in young females and can lead to severe medical complications affecting all body systems [2, 3]. The neurobiological processes underlying BN are unclear. Several studies have provided evidence that BN and other eating disorders are associated with structural and functional changes in brain regions, as detected using magnetic resonance imaging (MRI) (Table 1). For example, studies based on voxel-based morphometry (VBM) from MRI images have associated BN with increased grey matter volume (GMV) in the medial orbitalfrontal cortex and the ventral striatum [4], as well as in the insula [5], lingual gyri and inferior parietal lobule [6]. These and other studies also found reduced GMV in caudate and putamen [5, 7], bilateral medial frontal, precentral gyri, right postcentral gyrus, left superior (SFG) and inferior frontal gyri [6]. Some brain structural studies have found cortical abnormalities in BN, such as reduced cortical thickness in the bilateral frontal, temporal and parietal lobes, negtively related to the severity of BN symptoms [8]; and reduced cortical thickness in the right pars triangularis, right superior parietal and left dorsal posterior cingulate cortices as well as greater cortical thickness in the left ventral posterior cingulate cortex [9]. These and other brain structural studies have found abnormal structural connectivity in BN. Greater structural connectivity has been found among the insula, orbitofrontal cortex and ventral striatum, as well as among the bilateral frontal, temporal and parietal lobes. Conversely, lower connectivity has been found in the orbitofrontal cortex, amygdala and hypothalamus [9, 10]**.**

**Table 1:** A review table of the existing structural work in bulimia nervosa

Authors&Journal	Participants	Mean Age (SD)	Method	Findings	
				Regional Differences	Total Volume Difference
Schäfer et.al (2010) [4] Neuroimage. 2010;50 (2):639–43	BN(*n* = 14) BED(*n* = 17) HC(*n* = 19)	BN:23.1 (3.8) BED:26.4 (6.4) HC:22.3 (2.6)	VBM	Increased GMV of the medial OFC and the ventral striatum in BN (BN > HC).	Not assessed
Frank et.al(2013) [5] Am J Psychiatry. 2013;170 (10):1152–60	AN(n = 19) Recovered AN(n = 24)BN(*n* = 20) HC(*n* = 24)	AN:23.1 (5.8) Recovered AN:30.3 (8.1) BN:25.2 (5.3) HC:27.4 (6.3)	VBM	Increased GMV in the Left orbitofrontal cortex and anterior ventral insula (BN > HC);reduced GMV in the bilateral dorsal caudate and dorsal putamen in BN (BN < HC).	There was no significant difference between BN and HC in GMV, WMV and TIV(*P* > 0.05).
Coutinho et.al(2015) [7] Int J Eat Disord. 2015;48 (2):206–14	BN(*n* = 21) HC(n = 20)	BN:31.57 (8.27) HC:30.90 (8.79)	VBM	Reduced volume of caudate nucleus in BN (BN < HC).	There was no significant difference between BN and HC in TIV(P > 0.05).
In this research	BN(n = 34) HC(n = 34)	BN:22.85 (3.89) HC:22.26 (2.53)	VBM	Reduced GMV in the SFGmed. L, STG. R, DCG. R, DCG. L and SFGdor. L (BN < HC).	There were no significant differences between patients and controls in total GMV, WMV or TIV(P > 0.05).
Marsh et.al(2015) [6] Biol Psychiatry. 2015;77 (7):616–23	BN(n = 34) HC(n = 34)	BN:21.6 (6.0) HC:22.08 (6.5)	Morphological analysis including VBM and Cortical Thickness	Increased GMV in lingual gyri and inferior parietal lobule in BN (BN > HC); reduced GMV in bilateral medial frontal and precentral gyri, left superior and inferior frontal gyri, the right postcentral gyrus and bilateral temporoparietal areas (BN < HC).	The TIV did not differ across the BN and control group(P > 0.05).
Frank et.al(2016) [10] Transl Psychiatry. 2016;6 (11):e932	AN(*n* = 26) BN(*n* = 25) HC(n = 26)	AN:23.23 (5.26) BN:24.64 (4.22) HC:24.39 (3.49)	Structural and effective connectivity	Greater structural connectivity in insula, orbitofrontal cortex and ventral striatum (BN > HC), but lower connectivity in orbitofrontal cortex, amygdala and hypothalamus in BN (BN < HC).	Not assessed
Berner et al.(2018) [9] J Psychiatry Neurosci. 2018;43 (3):151–160	BN(*n* = 28) HC(n = 21)	BN:18.8 (4.1) HC:19.2 (5.5)	Cortical thickness	Greater cortical thickness in the left ventral posterior cingulate cortex in BN (BN > HC); reduced thickness of the right pars triangularis, right superior parietal and left dorsal posterior cingulate cortices (BN < HC).	Not assessed
Westwater et.al(2018) [8] Psychiatry Res Neuroimaging. 2018;271:118–125	BN(*n* = 37)	BN:22.6 (4.1)	Cortical thickness and structural connectivity	The cortical thickness in the left middle frontal gyrus, right superior frontal gyrus and bilateral orbitofrontal cortex (OFC) and temporoparietal regions negtively related to the severity of BN symptoms. The structural connectivity in the left OFC and middle temporal cortex negtively related to the severity of symptoms, while in the right superior parietal lobule positively related to the severity of symptoms.	Not assessed

These various studies have given inconsistent findings, likely reflecting small samples, differences in patient populations, and differences in methodology. For example, some studies corrected for age and total intracranial volume (TIV), while others corrected for age and body mass index (BMI). Several studies did not consider potential effects of medication. Many studies have not examined whether structural changes in the brain correlate with low self-esteem and self-evaluation, which are core features of BN and other eating disorders [11, 12].

The present study aimed to help clarify the potential association of BN with altered GMV in the brain, and to examine whether these structural changes correlate with patient’s self-evaluation. We retrospectively examined a small population of Chinese women with BN at our medical center, comparing them to a matched group of healthy controls.

## Methods

### Participants

This retrospective study involved 34 women diagnosed with BN at the Mental Health Center of West China Hospital, Sichuan University. The patients had engaged in binge eating and compensatory behaviors at least once a week during the three months prior to enrollment in the study. As controls, we recruited 34 age-matched women with BMI within the normal range of 18.5–23.9 who reported no history of eating disorders or other psychiatric disorders. Controls were recruited from the community through public advertisements. This study was approved by the Ethics Committee of West China Hospital, and all subjects provided written informed consent.

Patients and controls were assessed by two psychiatrists using the structured clinical interview for DSM-V disorders (SCID). All study subjects were right-handed, and none was currently taking medications or reported a history of major psychiatric disorders, head trauma, substance abuse or dependence, or neurologic disease. None of the subjects reported having metal implants or a pacemaker. Nine BN patients had mild anxiety and depressive symptoms based on the SCID, but we did not exclude them because these symptoms occur often in BN [13, 14].

### Clinical psychological assessment of patients

All patients completed the revised Chinese version of the Core Self-Evaluation Scale (CSES) and the Chinese version of the Rosenberg Self-Esteem Scale (RSES). The revised Chinese CSES uses 10 items to assess core self-evaluation, and it shows acceptable reliability and validity [13, 15]. The survey applies a 5-point scoring method, and the total score ranges from 10 to 50 points. The national norm on the CSES is 36.05 ± 5.21 for Chinese undergraduate women [15], and CSES scores of our patients were compared to this norm.

The RSES assesses self-esteem [16] and includes 10 items, with items 3, 5, 8, 9, 10 negatively worded. Each item is scored on a 4-point scale, and the individual item scores are summed to obtain a total score. We compared the RSES scores of our patients to the mean RSES score of 28.73 ± 4.48 reported for undergraduates in Beijing [17]. This norm did not vary significantly with gender in that study.

### MRI data acquisition

MR images were obtained using a Philips 3.0 T system. All subjects underwent 3D T1-weighted volumetric scanning with the following parameters: orientation, sagittal; matrix size, 256 × 256; field of view (FOV), 256 × 256 mm; slice thickness, 1 mm; gap, none; flip angle, 7°; repetition time (TR), 8.2 ms; echo time (TE), 3.8 ms; voxel size, 1 × 1 × 1 mm. During MRI, all subjects were asked to relax, keep their eyes closed without falling sleep and their head as motionless as possible, and not to think of anything in particular.

### VBM

MRI data were analyzed using SPM8 (Wellcome Trust Centre for Neuroimaging, Institute of Neurology, London, UK) and the VBM toolbox VBM8 (www.neuro.uni-jena.de/vbm/), both run within Matlab R2013b. Structural images were normalized to Montreal Neurological Institute (MNI) standard space and segmented into grey matter (GM), white matter (WM) and cerebrospinal fluid (CSF) using the unified segmentation approach. Finally, grey matter images were smoothed using an 8-mm full-width half-maximum Gaussian kernel.

### Statistical analysis

GMV in different brain regions was compared between patients and controls using the two-samples *t* test in SPM8. Covariates were age and TIV, defined as the sum of GM, WM and CSF volumes. Multiple comparisons were corrected using the family-wise error approach at a cluster level, with a corrected threshold of *P* < 0.001. An absolute threshold was set at 0.2 in all computations.

The two-samples *t* test in SPSS 20.0 (IBM, Chicago, IL, USA) was used to compare patients and controls in terms of clinico-demographic data as well as total GM, WM, CSF and intracranial volumes. Regions of interest (ROIs) showing GMV changes were defined using the “region of interest extraction” tool in xjView (www.alivelearn.net/xjview8/) via the SPM toolbox. Pearson correlation analysis was conducted within SPSS to explore relationships between ROIs and clinical variables after Bonferroni correction, with significance defined as *P* < 0.01.

## Results

### 3Clinico-demographics characteristics of participants

Table 2 summarizes the demographics and clinical characteristics of patients and healthy controls. The groups did not differ significantly in age, BMI or years of education (*P* > 0.05). Patients showed lower mean RSES and CSES scores than the corresponding norms from healthy populations (see Methods).

**Table 2:** Demographic variables,clinical characteristics and whole brain tissue volumes of the subjects

Characteristic	BN (*N* = 34) Mean ± SD	HC (*N* = 34) Mean ± SD	*P*
Age	22.85 ± 3.89	22.26 ± 2.53	NS
BMI (kg/m^2^)	20.46 ± 2.80	20.52 ± 1.52	NS
Duration of illness (years)^a^	2.92 ± 2.35	–	–
Education (years)	15.53 ± 2.43	16.09 ± 2.45	NS
CSES global score^a^	33.24 ± 3.56	–	
RSES global score^a^	25.76 ± 5.47	–	
Whole brain tissue volumes (VBM)			
Grey matter volume (ml)	660.56 ± 34.17	662.97 ± 37.11	NS
White matter volume (ml)	525.68 ± 39.46	526.02 ± 48.38	NS
CSF volume (ml)	227.69 ± 22.82	212.68 ± 24.09	< 0.05
Total intracranial volume (ml)	1413.94 ± 78.41	1401.67 ± 91.04	NS

*NS* not significant, *BMI* body mass index;

*CSES* the Core Self-Evaluation Scale, *RSES* the Rosenberg Self-Esteem Scale

^a^at the time of scaning

### VBM

#### Whole brain tissue volumes

There were no significant differences between patients and controls in total GMV, WMV or TIV (P > 0.05, Table 2). In contrast, patients showed a larger total CSF volume.

#### Regional GMV

**Fig. 1:**
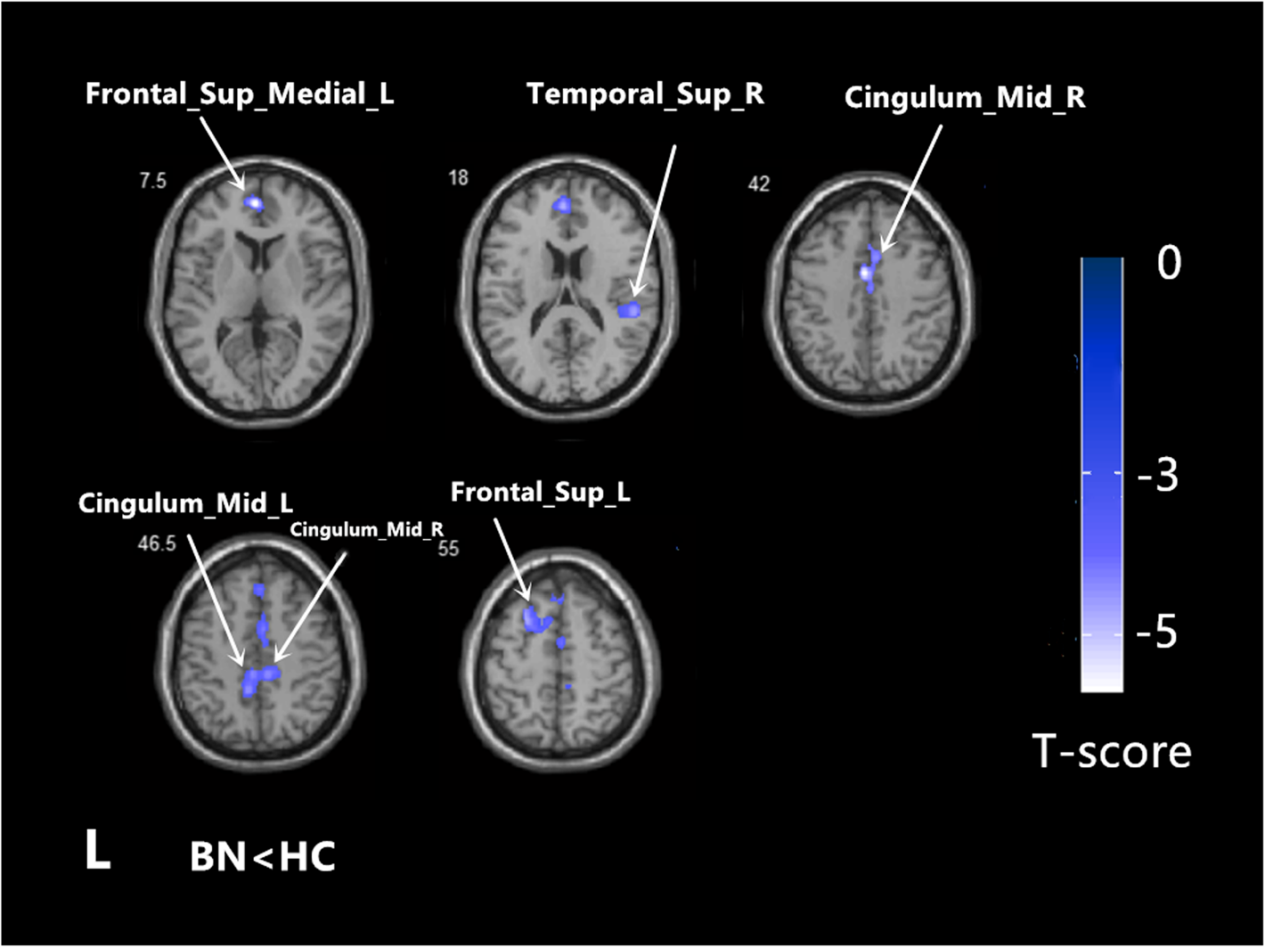
Regional grey matter volume alterations in BN (*n* = 34) relative to HC (*n* = 40). The statistical brain maps showed regional GMV decrease in BN, including the SFGmed. L, the STG. R, DCG. L, DCG. R and SFGdor. L (*P* < 0.001,corrected at cluster level). The color bar represents the t-scores

**Fig. 2:**
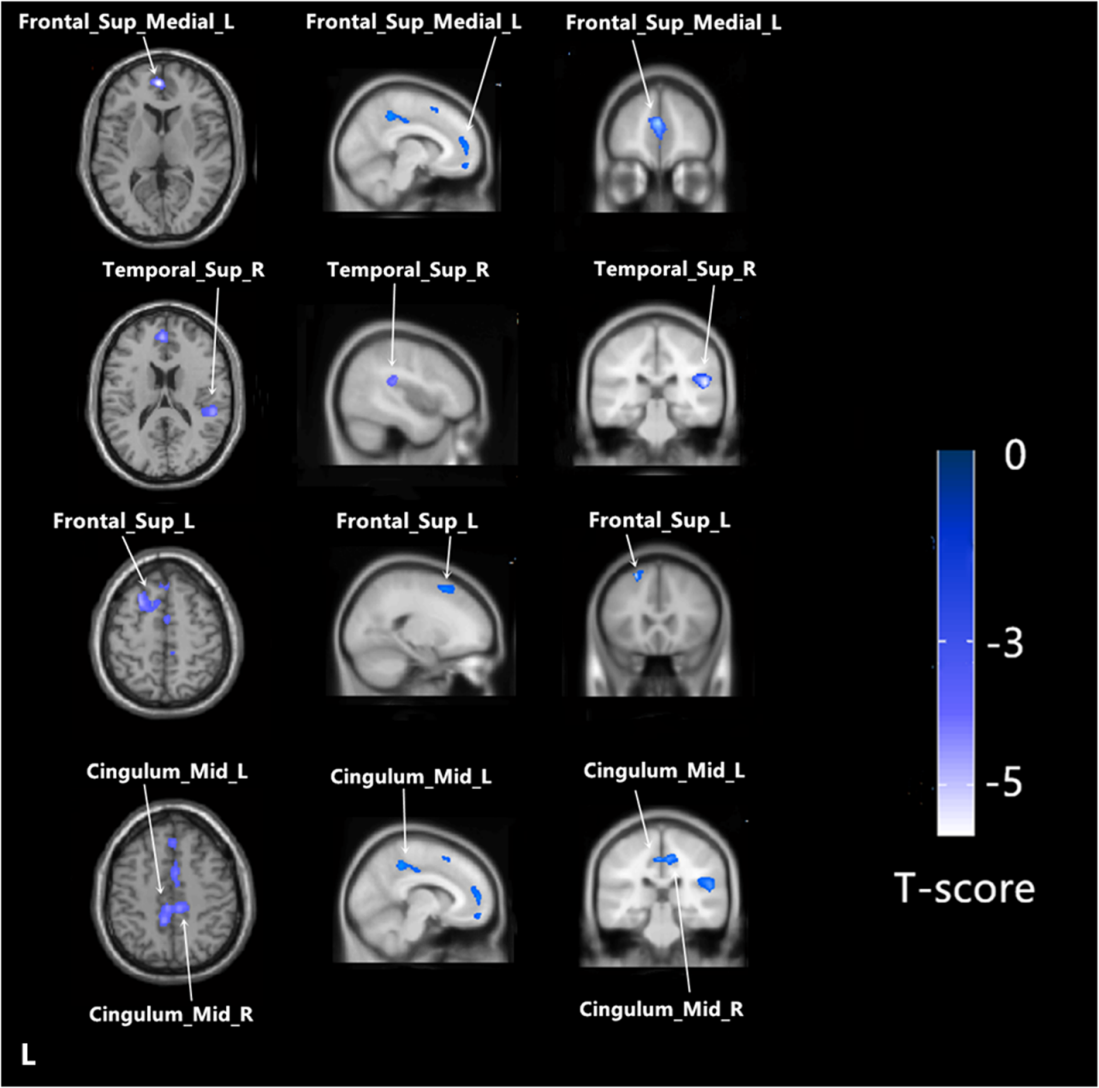
The sagittal/transvere/coronal views for each cluster (P < 0.001,corrected at cluster level). The color bar represents the t-scores

Using age and TIV as confounding covariates in VBM analysis, we identified five clusters of regional GMV after correction for multiple comparisons at the cluster level (threshold *P* < 0.001). Patients showed lower GMV in the left medial superior frontal gyrus (SFGmed.L), right superior temporal gyrus (STG.R), right median cingulate and paracingulate gyri (DCG.R), left median cingulate and paracingulate gyri (DCG.L) and left dorsolateral superior frontal gyrus (SFGdor.L) (Table 3 and Figs. 1 and 2). In none of the regions did patients show increased GMV.

**Table 3:** Regional GMV changes in patients with BN compared to healthy controls

Regions	Hemisphere	Cluster size (voxels)	Number of voxels within the anatomical region	*P*-value (FWE cluster-level)	Peak t values	MNI coordinates (x,y,z)
BN < HC						
Cluster 1		1704		< 0.001	−5.73	−1.5,52.5,7.5
Frontal_Sup_Medial_L	Left		855			
Cluster 2		767		< 0.001	−4.57	52.5,-33,18
Temporal_Sup_R	Right		347			
Cluster 3		876		< 0.001	− 4.85	−1.5,1.5,42
Cingulum_Mid_R	Right		364			
Cluster 4		1042				
Cingulum_Mid_L	Left		512	< 0.001	−4.42	−6,-42,46.5
Cluster 5		705				
Frontal_Sup_L	Left		366	< 0.001	−4.69	−24,18,55.5

### Correlations of GMV reduction with patient characteristics

**Fig. 3:**
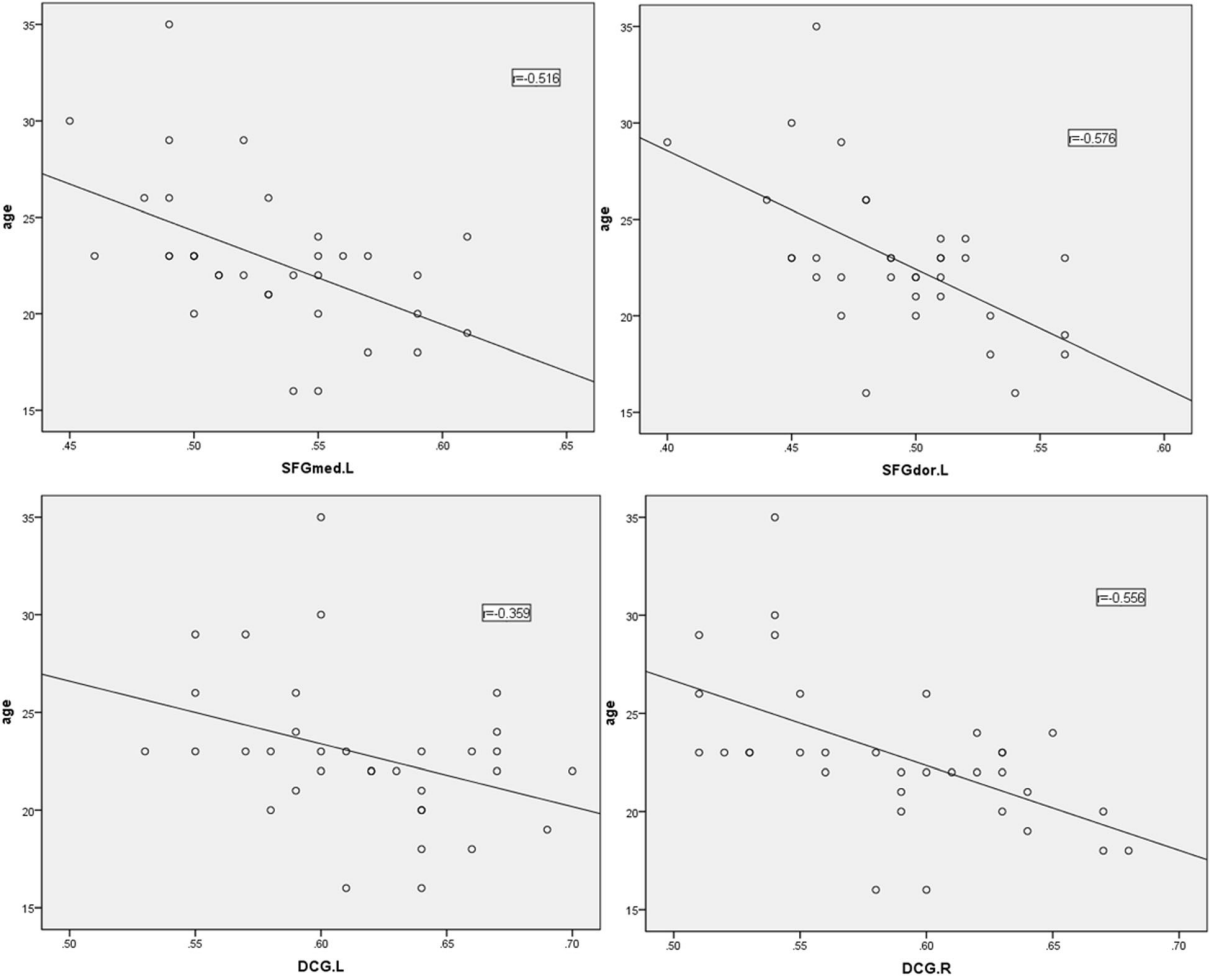
Correlation of regional grey matter volume with age in BN patients

BN-associated GMV reductions did not vary significantly with BMI, duration of illness, or total scores on the RSES or CSES (*P* > 0.05). In contrast, age did correlate negatively with GMV in the SFGmed. L (r = − 0.516, *P* < 0.005), DCG. R (r = − 0.556, P < 0.005), DCG. L (r = − 0.576, *P* < 0.05) and SFGdor. L (r = − 0.576, P < 0.005) (See Fig. 3).

## Discussion

### Global brain volume alterations

We found no significant differences between BN patients or controls in total GMV, WMV or intracranial volume, which is consistent with some previous studies in female patients [18, 19]. Our patients did show increased CSF volume, consistent with results obtained with one group of German patients [20] but not with a second German group [18]. These differences may reflect differences in methodology. Our observation of increased CSF volume but unchanged TIV suggests that the reduced GMV in BN is replaced by increased CSF.

### Regional GMV alterations

Patients showed reduced GMV in the SFGmed. L, STG. R, DCG. L, DCG. R and SFGdor. L, and no regions with increased GMV. These results are consistent with some studies but not with others, which have reported reduced GMV in the superior frontal gyrus and cingulate cortices [6], increased GMV in the medial orbital frontal cortex and the ventral striatum [4], or increased GMV in the paracentral lobule, precuneus, left putamen and insula, but reduced GMV in the caudate nucleus and thalamus [19]. Thus, different studies of BN patients vary considerably in the regions showing GMV alterations and the direction of the alteration (increase or decrease). These discrepancies may be due to differences in disease course, disease stage, medication history, ethnicity and other factors. Future studies should strive to examine larger, multi-center populations that may help reduce the influence of clinico-demographic factors on VBM.

The SFG is located in the superior part of the prefrontal cortex, which plays essential roles in executive control [21], including self-regulation of eating behavior [22]. The structural and functional abnormalities in the SFG in patients with BN or other eating disorders have been associated with deficits in self-regulation and reward processing [23]. Functional MRI studies have shown the SFG to be deactivated in BN in the presence of the expected reward during a reward-based learning task [24], during a Simon spatial incompatibility task [23]. The prefontal cortex is an important part of the fronto-striatal circuits, which is involved in self-regulatory control. Previous study has found deficient activity in the fronto-striatal circuits associated with impaired self-regulatory processes [23, 25]. The prefrontal cortex is also an important component of the forebrain system, which may contribute to eating dysregulation by driving maladaptive over- and undereating [26]. These alterations are likely lead to binge eating behaviors, thereby contributing to the development and maintenance of BN.

Reduced STG volume may cause abnormal body image perception and excessive concern about body shape and weight, leading to restrictive or binge eating. The GMV reduction in the STG in the Japanese study was associated with body dissatisfaction [27]. A functional MRI study has shown deactivation in STG when the individual was thinking about eating food [28]. Patients with eating disorders such as BN typically show emotional symptoms such as depressive and anxiety disorders [14, 29]. How these symptoms relate to inappropriate eating behaviors is controversial. Many studies have suggested that inappropriate eating behaviors are maladaptive strategies to manage negative feelings [30]. However, other studies have suggested that the eating behaviors alter mental state, such as by activating serotonin projections of the dorsal raphe to the prefrontal cortex; as a result, normalizing eating behavior can normalize psychiatric symptoms [31]. We found reduced GMV volume in the STG of our BN patients, which may link to more severe symptoms of depression and anxiety disorders [32, 33]. Such a reduction has also been linked to risk of suicide attempts in community samples of adolescents [34]. Further studies should examine whether such GMV reductions precede or follow the onset of inappropriate eating behaviors.

We observed reduced GMV in the middle cingulate cortex, and such a reduction has been rarely reported in BN. The middle cingulate cortex is associated with inhibitory functions and self-control, GMV in the middle cingulate cortex negtively correlated with uncontrlled eating behaviors has been reported in normal-weight female undergraduate students [35]. In addition, obese individuals in one study showed reduced activation of the middle cingulate cortex during appetite control [36]. The structural and functional alterations of the middle cingulate cortex in BN patients should be explored further.

### Correlations between GMV alterations and clinical variables

BN-associated GMV reductions did not vary significantly with BMI, duration of illness, or total scores on the RSES or CSES. Age did, however, correlate negatively with GMV in the SFGmed. L, DCG. R, DCG. L and SFGdor.L.

As expected, RSES and CSES scores were lower in BN patients than in controls, reflecting that a frequent symptom of BN is body image dissatisfaction [37], which is linked to low self-esteem [38, 39]. At the beginning of this study, we hypothesized that the RSES and CSES scores of BN patients would be associated with GMV alterations. However, we did not observe such an association in our patients. In contrast, a study of BN patients in the US did detect an association between brain functional alterations and self-esteem: activation of the right temporoparietal junction, precuneus and dorsal anterior cingulate cortex was weaker in BN patients than in controls during execution of social and self-knowledge tasks [40]. These brain regions are associated with self-knowledge and social processing. The discrepancy between that study and ours may be due to methodological differences. Whether the GMV reductions in our BN patients correlate with functional changes affecting self-esteem and self-evaluation should be explored further.

### Study limitations

This study analyzed only women, even though some men are also affected by eating disorders. In addition, some of our patients had mild anxiety and depressive symptoms, which may have confounded our results but which may also make our sample more representative of typical BN patient populations [14]. The cross-sectional nature of our study means that further work is needed to clarify whether the observed GMV alterations are the result of BN pathology or the consequence of binge-eating and compensatory behaviors. Our results should be verified and extended with much larger samples. In our study, we explored only brain structural alterations in BN; future work should explore changes in brain functional connectivity and networks in order to understand how brain areas interact with one another.

## Conclusions

The present study showed regional GMV decreases in several brain areas of women with BN. These structural alterations may be associated with impaired inhibitory control, body dissatisfaction, and emotion dysrugulation. While these GMV reductions correlated with patient age, they did not correlate with self-esteem or self-evaluation. These findings should be verified and extended in larger, preferably longitudinal studies.

## Data Availability

The datasets analyzed during the current study are available from the corresponding author on reasonable request.

## Abbreviations

AN: Anorexia nervosa
BMI: Body mass index
BN: Bulimia nervosa
CSES: Core Self-Evaluation Scale
CSF: Cerebrospinal fluid
DCG.L: Left median cingulate and paracingulate gyri
DCG.R: Right median cingulate and paracingulate gyri
GM: grey matter
GMV: Grey matter volume
MRI: Magnetic resonance imaging
ROI: Region of interest
RSES: Rosenberg Self-Esteem Scale
SCID: Structured clinical interview for DSM-V disorders
SFGdor.L: Left dorsolateral superior frontal gyrus
SFGmed.L: Left medial superior frontal gyrus
SPSS: Statistical Package for the Social Sciences
STG.R: Right superior temporal gyrus
TE: Echo time
TIV: Total intracranial volume
TR: Repetition time
VBM: Voxel-based morphometry
WM: White matter
WMV: White matter volume
